# Prognostic factors and failure patterns in non-metastatic nasopharyngeal carcinoma after intensity-modulated radiotherapy

**DOI:** 10.1186/s40880-016-0167-2

**Published:** 2016-12-28

**Authors:** Yan-Ping Mao, Ling-Long Tang, Lei Chen, Ying Sun, Zhen-Yu Qi, Guan-Qun Zhou, Li-Zhi Liu, Li Li, Ai-Hua Lin, Jun Ma

**Affiliations:** 1 State Key Laboratory of Oncology in South China, Collaborative Innovation Center for Cancer Medicine, Sun Yat-sen University Cancer Center, Guangzhou, 510060 Guangdong P. R. China; 2 Department of Radiation Oncology, Sun Yat-sen University Cancer Center, Guangzhou, 510060 Guangdong P. R. China; 3 Imaging Diagnosis and Interventional Center, Sun Yat-sen University Cancer Center, Guangzhou, 510060 Guangdong P. R. China; 4 Department of Medical Statistics and Epidemiology, School of Public Health, Sun Yat-sen University, Guangzhou, 510080 Guangdong P. R. China

**Keywords:** Nasopharyngeal carcinoma, Intensity-modulated radiotherapy, Prognosis, Failure pattern, Tumor staging

## Abstract

**Background:**

The prognostic values of staging parameters require continual re-assessment amid changes in diagnostic and therapeutic methods. This study aimed to identify the prognostic factors and failure patterns of non-metastatic nasopharyngeal carcinoma (NPC) in the intensity-modulated radiotherapy (IMRT) era.

**Methods:**

We reviewed the data from 749 patients with newly diagnosed, biopsy-proven, non-metastatic NPC in our cancer center (South China, an NPC endemic area) between January 2003 and December 2007. All patients underwent magnetic resonance imaging (MRI) before receiving IMRT. The actuarial survival rates were estimated using the Kaplan–Meier method, and survival curves were compared using the log-rank test. Multivariate analyses with the Cox proportional hazards model were used to test for the independent prognostic factors by backward eliminating insignificant explanatory variables.

**Results:**

The 5-year occurrence rates of local failure, regional failure, locoregional failure, and distant failure were 5.4, 3.0, 7.4, and 17.4%, respectively. The 5-year survival rates were as follows: local relapse-free survival, 94.6%; nodal relapse-free survival, 97.0%; distant metastasis-free survival, 82.6%; disease-free survival, 75.1%; and overall survival, 82.0%. Multivariate Cox regression analysis revealed that orbit involvement was the only significant prognostic factor for local failure (*P* = 0.011). Parapharyngeal tumor extension, retropharyngeal lymph node involvement, and the laterality, longest diameter, and Ho’s location of the cervical lymph nodes were significant prognostic factors for both distant failure and disease failure (all *P* < 0.05). Intracranial extension had significant prognostic value for distant failure (*P* = 0.040).

**Conclusions:**

The key failure pattern for NPC was distant metastasis in the IMRT era. With changes in diagnostic and therapeutic technologies as well as treatment modalities, the significant prognostic parameters for local control have also been altered substantially.

## Background

Nasopharyngeal carcinoma (NPC) is a challenge in oncology. Due to the deep-seated anatomic location and proximity of NPCs to critical structures, radical surgical resection is extremely difficult [1]. The introduction of radiotherapy has made this otherwise lethal malignant disease curable [1]. However, radiotherapy has undergone several different periods of development, and it was not until the advent of mega-voltage machines that a 5-year overall survival (OS) rate of 25% was first achieved, which marked the first major breakthrough in the treatment of NPC and established radiotherapy as the primary modality of choice for NPC [2].

Since that time, progressive improvements in the treatment outcomes of patients with NPC have been achieved. Initially, in the 1970–1980s, conventional radiotherapy alone resulted in similar outcomes in both the endemic and non-endemic areas, with 5-year OS rates of 48%–52%, a cumulative local failure rate of 20%, a cumulative regional failure rate of 14%, and a cumulative distant metastasis rate of 19% [3, 4]. During the 1990s, rapid technological advances in imaging methods, computerized planning systems, and radiotherapy facilities and the accumulation of radiobiological knowledge that enabled schedule, dose, and fractionation optimization led to better outcomes after radiotherapy, with 5-year OS rates of 65%–74%, a cumulative local failure rate of 12%, a regional failure rate of 5%, and a distant metastasis rate of 16% [5, 6]. However, local relapse and distant metastasis remained the two major causes of failure. Intensity-modulated radiotherapy (IMRT) was introduced in the 2000s and represented a major breakthrough in the radiotherapeutic management of NPC. Due to the dosimetric advantages of IMRT combined with the use of magnetic resonance imaging (MRI)-guided tumor volume delineation and concurrent chemotherapy, local control rate in NPC patients has improved significantly [7, 8].

With these changes in diagnostic and therapeutic technologies, the significant prognostic parameters that have been identified in patients treated with conventional radiotherapy may also have been altered substantially. Therefore, caution must be applied when applying prognostic factors to predict prognosis and guide treatment strategies in patients treated using these modern, more effective treatment modalities [9]. Thus, the prognostic values of staging parameters require continual re-assessment amid these changes in diagnostic and therapeutic methods. However, few studies have systematically investigated the prognostic factors in NPC in the IMRT era. We performed a retrospective systematic evaluation of a large cohort of patients with NPC who were treated with IMRT at the Sun Yat-sen University Cancer Center in South China, an NPC endemic area. The aim of this study was to identify the key failure patterns and prognostic factors to improve the treatment of patients with NPC and provide a baseline for future studies.

## Methods

### Patient cohort

We reviewed the medical records of all patients with NPC treated with IMRT at the Sun Yat-sen University Cancer Center between January 2003 and December 2007. We included all patients with newly diagnosed, non-metastatic, histologically confirmed disease. All patients were staged using a standard protocol [10] comprising a complete disease history, physical examination, hematology and biochemistry profiles, fiberoptic nasopharyngoscopy, MRI of the neck and nasopharynx, chest radiography, abdominal sonography, and a whole-body bone scan using single-photon emission computed tomography (SPECT). All MRI materials and clinical records were reviewed to minimize heterogeneity in restaging. The study was reviewed and approved by the ethics committee of the Sun Yat-sen University Cancer Center.

### Image assessment and criteria for staging parameters

Two radiologists specializing in head and neck cancers evaluated all scans separately. All parameters included in the TNM staging were re-assessed, and any disagreements were resolved by consensus. Parapharyngeal tumor extension was identified as a direct tumor extension through the pharyngobasilar fascia [11]. Nasal cavity extension was defined as tumor extension beyond the bony nasal septum [12]. Oropharyngeal extension was defined as the primary tumor extension of the mucosa or submucosal tissue below the plane of the superior surface of the soft palate or the C1–C2 interspace [13]. Hypopharyngeal extension was defined as the primary tumor extension of the mucosa or submucosal tissue to the superior border of the epiglottis or lower margin of the C3 interspace [14]. The skull base included the clivus, pterygoid bones, body of the sphenoid, apices of the petrous temporal bones, sphenoid wings, upper cervical spine, and skull base foramina and fissures [15]. The criterion for orbit extension was tumor extension into the orbital apex, inferior orbital fissure, or superior orbital fissure [16]. Involvement of the cavernous sinus, brain tissue, cistern, or dural meninges was interpreted as intracranial extension [17].

The diagnostic criteria for metastatic lymphadenopathy [18] include the following: (a) lateral retropharyngeal lymph nodes with a minimal axial diameter in the largest plane of an individual node of at least 5 mm and any node observed in the median retropharyngeal group, lymph nodes with a minimal axial diameter of at least 11 mm in the jugulodigastric region and 10 mm for all other cervical nodes; (b) lymph nodes of any size with central necrosis or a contrast-enhanced rim; (c) nodal groups, defined as the presence of three or more contiguous and confluent lymph nodes, each of which with a minimal axial diameter of 8–10 mm; and/or (d) lymph nodes of any size with extracapsular spread, the presence of indistinct nodal margins, irregular nodal capsular enhancement, or infiltration into the adjacent fat or muscle.

### Treatment

All patients underwent radical radiotherapy. The nasopharyngeal and upper neck tumor volumes were treated using IMRT for the entire treatment course. The lower neck was treated with a conventional anterior or anteroposterior opposing cervical technique. The prescribed doses were 68 Gy to the planning target volume of the primary tumor (PTV-P), 60–68 Gy to the PTV of the nodes (PTV-N), 60 Gy to the high-risk regions (PTV1), and 54 Gy to the low-risk regions (PTV2) in 30 fractions over 6 weeks. The anterior split-neck field received 50–60 Gy irradiation in 2-Gy fractions. The irradiation was delivered once daily for 5 days per week. Further details of the IMRT techniques used at our cancer center have been previously reported [10].

During the study period, concurrent chemoradiotherapy was recommended for stage III to IVa-b disease, and no chemotherapy was recommended for stage I to IIa disease (as defined using the 6th edition of the International Union against Cancer/American Joint Committee on Cancer [UICC/AJCC] staging system). However, some patients with stage III to IVa–b disease also received neoadjuvant or adjuvant chemotherapy, and some patients with stage IIb disease received concurrent chemoradiotherapy. Neoadjuvant or adjuvant chemotherapy consisted of cisplatin (80–100 mg/m^2^) with either 5-fluorouracil (800–1000 mg/m^2^) or taxanes (paclitaxel [135–175 mg/m^2^] or docetaxel [70–75 mg/m^2^]) every 3 weeks for 3 cycles. Concomitant chemotherapy consisted of cisplatin (80–100 mg/m^2^) given on weeks 1, 4, and 7 of radiotherapy or cisplatin (30–40 mg/m^2^) given weekly. When possible, salvage treatments, such as intracavitary brachytherapy, surgery, and chemotherapy, were provided in the event of documented relapse or persistent disease.

### Follow-up

Treatment responses and toxicity were assessed in each patient every week during treatment, every 2–3 months during the first 2 years after treatment, and then every 3–6 months during the following 3 years. Endoscopy, computed tomography (CT), or MRI scans of the head and neck were also performed every 3 months in the first year and annually thereafter. Additional tests were performed when indicated to evaluate local or distant failure. The last follow-up time was 30th August 2013.

### Statistical analysis

All analyses were performed using SPSS version 16.0 (SPSS, Chicago, IL, USA). The actuarial rates were estimated using the Kaplan–Meier method, and the survival curves were compared using the log-rank test. The following endpoints (measured from the start of treatment to the first defined event) were estimated: local relapse-free survival (LRFS), nodal relapse-free survival (NRFS), distant metastasis-free survival (DMFS), disease-free survival (DFS), and OS.

Multivariate analyses with the Cox proportional hazards model were used to test for independent prognostic factors by backward elimination of the insignificant explanatory variables. The Cox proportional hazards model was also used to calculate the hazard ratios (HRs) and 95% confidence intervals (CIs). Host factors (age and sex) were included as covariates in all tests. Two-tailed *P* values <0.05 were considered statistically significant.

## Results

### Patient characteristics

The median patient age was 43 years (range, 13–78 years), and the cohort included 580 males and 169 females (male:female ratio of 3.4:1). Histologically, 744 (99.3%) patients had World Health Organization (WHO) type II/III disease, and 5 (0.7%) had WHO type I disease. Positron emission tomography (PET)/CT was performed for 162 (21.6%) patients. Using the 2009 7th UICC/AJCC staging system, the numbers of patients with stage I, II, III, IVa, and IVb disease were 78 (10.4%), 179 (23.9%), 282 (37.7%), 160 (21.3%), and 50 (6.7%), respectively. The median follow-up duration was 81.4 months (range, 3.1–126.5 months). In total, 424 (86.2%) of the 492 patients with stage III–IV disease received chemotherapy. The characteristics of the patients are summarized in Table 1.

**Table 1:** Clinicopathologic features of the 749 patients with nasopharyngeal carcinoma (NPC)

Feature	No. of patients [cases (%)]
Sex	
Male	580 (77.4)
Female	169 (22.6)
WHO pathology	
Type I	5 (0.7)
Type II/III	744 (99.3)
Clinical stage^a^	
I–II	257 (34.3)
III–IV	492 (65.7)
T stage^a^	
T1	177 (23.6)
T2	140 (18.7)
T3	264 (35.2)
T4	168 (22.4)
N stage^a^	
N0	184 (24.6)
N1	409 (54.6)
N2	106 (14.2)
N3	50 (6.7)
Chemotherapy	
No chemotherapy	214 (28.6)
Concurrent	243(32.5)
Induction + concurrent	246 (32.8)
Concurrent + adjuvant	46 (6.1)

*WHO* World Health Organization

^a^According to the 7th edition of the Union for International Cancer Control/American Joint Committee on Cancer (UICC/AJCC) staging system

### Dose-volume histogram analysis

Table 2 summarizes the dose-volume histogram (DVH) statistics for the target volumes of interest. The maximum dose (D_max_) and minimum dose (D_min_) of radiation were defined as the maximum and minimum dose points in the DVH. The radiation dose prescribed to the PTV-P was 68 Gy for all patients. Because the proportion of the volume that received <93% of the prescribed dose was set to ≤3%, the actual median dose to the PTV-P was 73.34 Gy.

**Table 2:** Dose-volume histogram data for planning target volumes in the 749 patients with NPC

Variate	Volume (mL)	D_max_ (Gy)	D_min_ (Gy)	Mean dose (Gy)
PTV-P	51.89 (2.60–287.48)	79.91 (70.47–93.95)	59.79 (50.58–71.16)	73.11 (68.16–78.48)
PTV1	97.35 (13.78–494.77)	79.67 (72.81–86.08)	58.86 (49.13–67.32)	67.94 (56.66–74.48)
PTV2	368.97 (106.36–685.58)	78.99 (70.04–86.74)	45.28 (43.12–61.47)	60.55 (53.64–72.13)

*PTV*-*P* planning target volume of the primary tumor, *PTV1* PTV of the high-risk clinical target volume, *PTV2* PTV of the low-risk clinical target volume,* D*_*max*_ maximum dose,* D*_*min*_ minimum dose. All data are presented as median with range in parentheses

The dose limitations are ≤60 Gy to the brainstem and ≤50 Gy to the spinal cord. In some advanced diseases with extensive extension, we would relax the dose limitation to D_max_ ≤ 63 Gy. The median D_max_ for the brainstem was 54.16 Gy (range, 31.68–77.65 Gy), and the median proportion of the volume in the brainstem that received >60 Gy was 9.67% (range, 0.03%–26.97%). The median D_max_ for the spinal cord was 33.96 Gy (range, 8.29–60.54 Gy), and the median proportion of the volume in the spinal cord that received >50 Gy was 0.97% (range, 0.01%–3.42%). However, 3 of the 749 patients received irradiation with a D_max_ > 63 Gy, with no serious late toxicity for the brainstem. The median doses to the left and right parotid glands were 28.20 Gy (range, 11.90–49.70 Gy) and 29.98 Gy (range, 21.30–42.02 Gy). The median proportions of the volumes in the left and right parotid glands that received a dose >26 Gy were 49.34% (range, 9.61%–98.25%) and 51.23% (20.59%–96.61%).

### Patterns of treatment failure and survival

Among the 749 patients, 56 (7.5%) relapsed [34 (4.5%) had local relapse only, 15 (2.0%) had nodal relapse only, 7 (1.0%) had both local and nodal relapse], and 129 (17.2%) had distant metastasis. One hundred forty-nine (19.9%) patients died. The median time to relapse was 25.2 (range, 7.0–93.8) months, and the median time to distant metastases was 18.9 (range, 2.6–92.1) months.

**Fig. 1:**
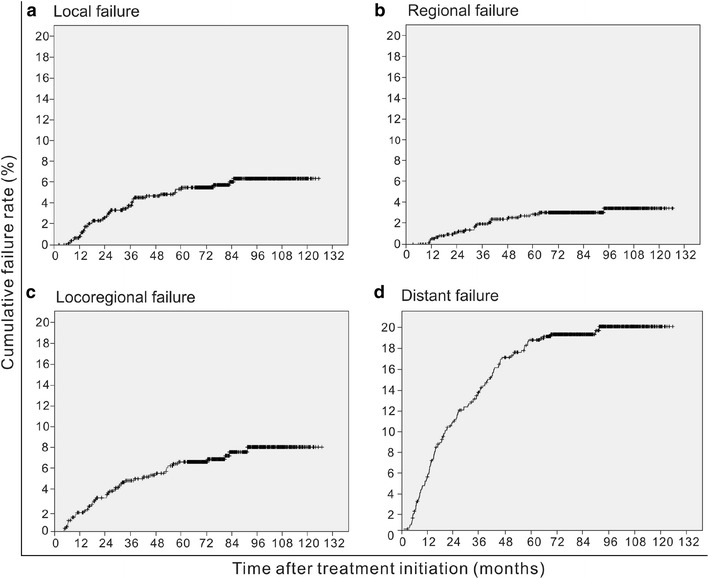
Cumulative local failure (**a**), regional failure (**b**), locoregional failure (**c**), and distant failure (**d**) rates of 749 patients with nasopharyngeal carcinoma (NPC) who were treated with intensity-modulated radiation therapy (IMRT)

**Fig. 2:**
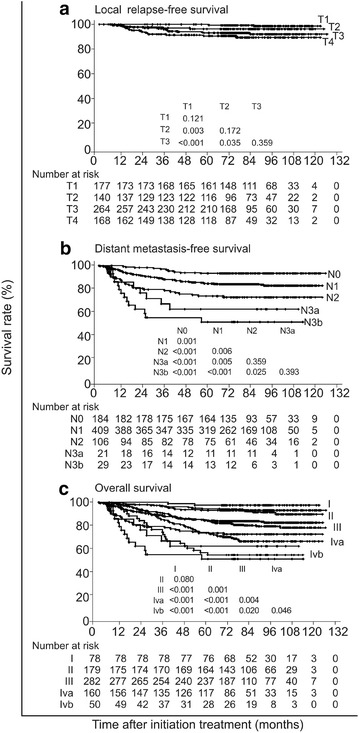
Survival curves of 749 patients with NPC who were treated with IMRT. The current 7th edition of the Union for International Cancer Control/American Joint Committee on Cancer (UICC/AJCC) staging system was used for staging. **a** The 5-year local relapse-free survival (LRFS) rates for patients with stage T1-T4 NPC were 98.4, 96.2, 93.0, and 90.5%, respectively. **b** The 5-year distant metastasis-free survival (DMFS) rates for patients with stage N0-N3b NPC were 93.3, 84.2, 72.4, 61.9, and 50.7%, respectively. **c** the 5-year overall survival (OS) rates for patients with stage I-IVb NPC were 97.4, 93.8, 81.8, 69.7, and 54.0%, respectively

The 5-year local failure, regional failure, locoregional failure, and distant failure rates for the whole cohort were 5.4, 3.0, 7.4, and 17.4%, respectively (Fig. 1). The 5-year survival rates for the whole cohort were as follows: LRFS, 94.6%; NRFS, 97.0%; DMFS, 82.6%; DFS, 75.1%; and OS, 82.0%. Using the 7th edition of the UICC/AJCC staging system, the 5-year LRFS rates for patients with stage T1–T4 NPC were 98.4, 96.2, 93.0, and 90.5%, respectively (Fig. 2a); the 5-year DMFS rates for patients with stage N0-N3b NPC were 93.3, 84.2, 72.4, 61.9, and 50.7%, respectively (Fig. 2b); and the 5-year OS rates for patients with stage I–IVb NPC were 97.4, 93.8, 81.8, 69.7, and 54.0%, respectively (Fig. 2c).

### Prognostic factors

Table 3 lists the factors with independent significance for different endpoints. The TNM stage was consistently the most significant factor for prognosis prediction: T stage for local failure prediction (*P* < 0.001), and both T and N stages for distant failure, disease failure, and death prediction (all *P* < 0.001). None of the factors had a significant influence on nodal failure. An age >50 years was associated with significantly higher rates of distant failure (HR = 1.530, 95% CI 1.037–2.256, *P* = 0.032), disease failure (HR = 1.896, 95% CI 1.403–2.561, *P* < 0.001), and death (HR = 2.482, 95% CI 1.770–3.482, *P* < 0.001).

**Table 3:** Multivariate analyses of significant factors for various endpoints in the 749 patients with NPC

Variable	Local failure		Regional failure		Distant failure		Disease failure		Death (any cause)	
	HR (95% CI)	*P* value	HR (95% CI)	*P* value	HR (95% CI)	*P* value	HR (95% CI)	*P* value	HR (95% CI)	*P* value
T stage (T2, T3, and T4 vs. T1)	1.956 (1.371–2.790)	<0.001	1.243 (0.809–1.912)	0.321	1.556 (1.282–1.888)	<0.001	1.589 (1.363–1.853)	<0.001	1.583 (1.328–1.886)	<0.001
N stage (N1, N2, and N3 vs. N0)	1.233 (0.833–1.723)	0.218	1.416 (0.928–2.160)	0.106	1.741 (1.485–2.041)	<0.001	1.617 (1.415–1.847)	<0.001	1.690 (1.455–1.964)	<0.001
Age (>50 vs. ≤50 years)	1.011 (0.490–2.086)	0.976	1.005 (0.365–2.769)	0.992	1.530 (1.037–2.256)	0.032	1.896 (1.403–2.561)	<0.001	2.482 (1.770–3.482)	<0.001
Sex (female vs. male)	0.826 (0.378–1.802)	0.631	0.797 (0.266–2.386)	0.685	1.066 (0.689–1.649)	0.776	1.017 (0.713–1.450)	0.926	0.900 (0.587–1.379)	0.627
Histology (type II/III vs. type I)	1.930E4 (0.000–6.656E258)	0.974	2.104E4 (0.000–)	0.981	2.313E4 (0.000–4.202E155)	0.955	1.397 (0.194–10.043)	0.739	1.037 (0.144–7.480)	0.971
Chemotherapy (CCRT, induction with CCRT, and CCRT with adjuvant vs. none)	0.921 (0.705–1.202)	0.544	0.920 (0.629–1.345)	0.666	1.002 (0.870–1.155)	0.973	0.947 (0.840–1.068)	0.375	0.969 (0.847–1.108)	0.644

*HR* hazard ratio, *CI* confidence interval, *CCRT* concurrent chemoradiotherapy

The involvement of the nasal cavity, oropharynx, parapharynx, skull base, paranasal sinus, cranial nerve palsy, intracranial extension, infratemporal fossa, intracranial extension, orbit, and hypopharynx (all were T stage-related parameters) occurred in 280 (37.4%), 85 (11.3%), 540 (72.1%), 429 (57.3%), 36 (4.8%), 35 (4.7%), and 3 (0.4%) patients, respectively. To explore the prognostic values of the specific anatomic factors included in the TNM staging system, we analyzed all T stage-related parameters, all N stage-related parameters (i.e., retropharyngeal lymph node involvement as well as the laterality, longest diameter, and Ho’s location of the involved cervical lymph nodes), age, histological type, and chemotherapy modality using univariate and multivariate analyses. With the exception of hypopharyngeal involvement, the univariate analysis revealed that age, histological type, and all T stage- and N stage-related parameters were significantly associated with disease failure (all *P* < 0.05). All factors that were significant in univariate analysis were included in the Cox proportion hazards model with the backward elimination of non-significant explanatory variables. Only orbit involvement was found to be significant prognostic factor for local failure in the multivariate analysis (*P* = 0.011). Parapharyngeal involvement, retropharyngeal lymph node involvement, and the laterality, longest diameter, and Ho’s location of the involved cervical lymph nodes were significant prognostic factors for both distant failure and disease failure (all *P* < 0.05). Intracranial extension had significant prognostic value for distant failure (*P* = 0.040) (Table 4).

**Table 4:** Multivariate prognostic analyses of anatomic factors in the 749 patients with NPC

Variable	Disease failure		Local failure		Distant failure	
	HR (95% CI)	*P* value	HR (95% CI)	*P* value	HR (95% CI)	*P* value
Age (>50 vs. ≤50 years)	1.857 (1.365–2.516)	<0.001	0.996 (0.476–2.080)	0.997	1.433 (0.967–2.120)	0.074
Histological type (WHO II/III vs. WHO I)	1.371 (0.829–2.264)	0.221	0.843 (0.327–2.125)	0.710	3.614 (1.804–7.219)	<0.001
Chemotherapy (CCRT, induction with CCRT, and CCRT with adjuvant vs. none)	1.207 (0.794–1.843)	0.383	0.624 (0.273–1.410)	0.269	1.459 (0.834–2.569)	0.190
Nasal cavity involvement (yes vs. no)	0.946 (0.683–1.311)	0.741	0.867 (0.429–1.752)	0.690	0.818 (0.547–1.225)	0.330
Oropharyngeal involvement (yes vs. no)	1.208 (0.812–1.798)		0.431 (0.130–1.432)	0.169	1.491 (0.941–2.362)	0.089
Parapharyngeal involvement (yes vs. no)	1.843 (1.144–2.969)	0.011	2.212 (0.690–7.141)	0.172	2.040 (1.115–3.764)	0.017
Skull base involvement (yes vs. no)	1.273 (0.851–1.906)	0.240	2.113 (0.790–5.651)	0.136	1.248 (0.759–2.052)	0.383
Paranasal sinus involvement (yes vs. no)	1.078 (0.704–1.650)	0.729	1.246 (0.526–2.949)	0.617	0.937 (0.543–1.616)	0.815
Cranial nerve involvement (yes vs. no)	1.394 (0.867–2.178)	0.139	2.012 (0.833–4.881)	0.120	1.247 (0.703–2.218)	0.399
Intracranial involvement (yes vs. no)	1.303 (0.871–1.950)	0.198	1.119 (0.468–2.674)	0.800	1.538 (1.056–2.490)	0.040
Infratemporal fossa involvement (yes vs. no)	1.035 (0.564–1.875)	0.893	0.616 (0.175–2.137)	0.458	0.737 (0.320–1.675)	0.467
Orbit involvement (yes vs. no)	1.641 (0.914–2.937)	0.109	4.067 (1.369–12.103)	0.011	1.537 (0.733–3.231)	0.251
Retropharyngeal lymph node involvement (yes vs. no)	1.583 (1.102–2.273)	0.011	1.519 (0.679–3.406)	0.309	1.565 (1.078–2.499)	0.030
Cervical lymph node involvement						
Longest diameter (>6 cm vs. ≤6 cm)	2.829 (1.603–4.991)	<0.001	0.815 (0.107–6.219)	0.840	3.050 (1.625–5.727)	0.001
Laterality (bilateral vs. unilateral)	1.475 (1.031–2.107)	0.032	0.398 (0.672–6.513)	0.423	1.625 (1.078–2.453)	0.019
Ho’s location (Ho’s vs. above Ho’s)	2.396 (1.435–3.996)	0.001	0.567 (0.073–4.272)	0.583	2.859 (1.617–5.061)	<0.001

*HR* hazard ratio, *CI* confidence interval, *CCRT* current chemoradiotherapy

## Discussion

The present study, with 5-year LRFS and DMFS rates of 94.6% and 82.6%, confirmed that distant metastasis has surpassed local relapse to be the major cause of failure in NPC in the IMRT era, whereas our previous data from the conventional radiotherapy era demonstrated that both local relapse and distant metastasis were the major causes of failure in NPC, with 5-year LRFS and DMFS rates of 85% and 81% [6]. Although T stage was still a significant predictor for local failure, with the more extensive primary extension associated with poorer local control, the 5-year LRFS rates for patients with NPC at adjacent T stages were not significantly different. T stage is becoming less powerful in segregating patients into various risk groups. Improvements in local control have reduced the significance of known prognostic factors, even the T stage, for local control prediction [9]. It has been recommended that three rather than four T stages should be applied to classify patients in the modern era [19, 20]. In the future, treatment modality may be the only significant predictor for local control in NPC patients; and the most important challenge in the management of NPC should be the exploration of more effective systemic agents to control distant metastasis.

Based on CT and conventional radiotherapy, several studies have systematically investigated prognostic factors for local control in patients with NPC [21–23]. Two studies have demonstrated that skull base extension or cranial nerve palsy could affect local failure-free survival [21, 22], whereas one has confirmed that parapharyngeal tumor extension was an independent prognostic factor for local failure-free survival [23]. Based on MRI and IMRT, no systematic data referring to prognostic factors in patients with NPC were available. Zong et al. [24] focused on skull base extension and found that it did not influence local failure in the IMRT era. Based on the same database presented here, our previous studies reported that skull base extension [25] and parapharyngeal tumor extension [26] were not independent prognostic factors for local control in patients with NPC treated with IMRT. In the current study, we systematically re-evaluated all of the prognostic factors included in the TNM staging system and found that their prognostic values no longer remained significant and that orbit involvement was the only independent prognostic factor for local control.

Improvements in local control can explain these results. First, due to its improved dose conformability and escalation, IMRT eliminates the areas at the edges of the shielding blocks and field borders, such as the sphenoid sinus behind the pituitary shield, parapharyngeal region, and posterior skull base, which would be under-dosed when two-dimensional conventional radiotherapy is applied [27]. Thus, IMRT leads to improved local control in these areas. Second, MRI improves visualization and is superior to CT for identifying parapharyngeal tumor extension, skull base extension, and intracranial involvement [13]. Therefore, a more precise dose of radiation can be delivered to the gross target volume of primary tumor, which ultimately improves local control for these areas. However, orbit extension, which usually occurs as tumor extension from the pterygopalatine fossa via the inferior orbital fissure or directly from the cavernous sinus, is a marker of the most severe extension of NPC [15]. Even with the most sophisticated treatment techniques and intensive use of chemotherapy, some patients with advanced T4 disease that approaches important organs at risk cannot tolerate a radiation dose of 70 Gy, and the local control rate for this subgroup remains low, even after IMRT [28]. Some patients who had tiny intracranial extensions (for example, through the oval foramen or foramen lacerum to cavernous sinus) or cranial nerve palsy and did not have disease approaching important organs at risk were still able to tolerate a radiation dose of 70 Gy and achieve excellent local control. This finding might explain why orbit involvement alone and not intracranial extension or cranial nerve palsy was the only independent prognostic factor for local control.

In patients diagnosed using CT and treated with conventional radiotherapy, parapharyngeal tumor extension, skull base extension, cranial nerve palsy, retropharyngeal lymph node involvement, and all cervical lymph node-related parameters (i.e., laterality, size, Ho’s location, and fixity) were proved to be significant prognostic factors for distant control [22, 29]. In the present study, patients were diagnosed using MRI and treated with IMRT, and parapharyngeal tumor extension and all N stage-related parameters (i.e., retropharyngeal lymph node involvement as well as the laterality, size, and Ho’s location of involved cervical lymph nodes) remained prognostic significance for DMFS. A number of studies have confirmed that IMRT has improved local control but not distant control in NPC [8, 10, 28]. Thus, most of the parameters that influence distant control have not been altered by the introduction of IMRT.

Compared with previous studies, one major different finding in the present study is that intracranial extension rather than cranial nerve palsy was a significant prognostic factor for distant control. Intracranial extension, which was defined as the involvement of the cavernous sinus, brain tissue, cistern, or dural meninges and could not be easily detected by CT, could be demonstrated more precisely by MRI because of its better tissue contrast and multiplanar capacity [13]. It has been reported that most patients with clinical cranial nerve palsy display one or more sites of intracranial involvement on MRI [30]. Therefore, the previously demonstrated adverse prognostic significance of cranial nerve palsy may be accurately reflected by intracranial extension on MRI.

Another difference was the lack of significant prognostic value for skull base extension in terms of DMFS in the present study. It has been reported that 51.2% of patients with skull base extension shown on MRI but not on CT images had minimal bone disease [13]. These patients would have a more favorable prognosis [31, 32]. Although the diagnosis of skull base extension based entirely on MRI leads to an upstaging according to the UICC/AJCC staging system, skull base extension has not been confirmed as a significant prognostic factor for DMFS. However, the prognostic value of MRI-detected skull base extension varies in different studies; although MRI-detected skull base extension was not an independent prognostic factor, the grading of skull base extension according to the site of extension has previously been reported to be an independent prognostic factor for both OS and DMFS [33].

The prognosis of a patient with cancer depends on the biological aggressiveness of the tumor, host factors, and therapeutic interventions [9]. However, we explored the prognosis only according to anatomic factors in this study. An increasing number of biological, genetic, and molecular factors, such as plasma/serum Epstein–Barr virus (EBV) DNA [34] and specific microRNAs (miRNAs) [35], as well as the target volume [36, 37] and other non-anatomic factors, have been identified and studied [38]. Such factors may have profound influences on the prognosis for individual patients. However, the extent of local extension, regional lymphatic spread, and distant metastasis as reflected by TNM staging still remain the most important prognostic factors in NPC [9].

Because the definition of parapharyngeal tumor extension has differed substantially across different time points [26, 39], it is difficult to compare the prognostic value of parapharyngeal tumor extension. This was a retrospective study that aimed to evaluate the prognostic value of staging parameters amid the changes in diagnostic and therapeutic methods. Moreover, due to financial factors and some patients’ lack of health insurance, PET/CT was only performed on 130 (25.4%) of the 749 patients in our study. Additional effects of sensitive and specific imaging technologies such as PET on stage migration are expected. With its well-recognized superiority in the detection of nodal metastases and distant metastases [40, 41], there seems to be little doubt that the addition of PET (with or without CT) could contribute to improve staging accuracy and prognostication in NPC.

## Conclusions

The key failure pattern for NPC in the IMRT era was distant metastasis. With the changes in diagnostic and therapeutic technologies, the significant prognostic parameters for local control have also altered substantially. Orbit involvement was the only independent prognostic factor for local control, and intracranial extension, parapharyngeal tumor extension, and all N stage-related parameters were independent prognostic factors for DMFS.
